# Reinforcement in the banded darter *Etheostoma zonale*: The effect of sex and sympatry on preferences

**DOI:** 10.1002/ece3.6076

**Published:** 2020-02-12

**Authors:** Natalie S. Roberts, Tamra C. Mendelson

**Affiliations:** ^1^ Department of Biological Sciences University of Maryland, Baltimore County Baltimore MD USA

**Keywords:** behavioral isolation, *Etheostoma*, male mate choice, reinforcement, speciation

## Abstract

Reinforcement occurs when selection against hybrid offspring strengthens behavioral isolation between parental species and may be an important factor in speciation. Theoretical models and experimental evidence indicate that both female and male preferences can be strengthened upon secondary contact via reinforcement. However, the question remains whether this process is more likely to affect the preferences of one sex or the other. Males of polygynous species are often predicted to exhibit weaker preferences than females, potentially limiting the ability for reinforcement to shape male preferences. Yet, in darters (Percidae: *Etheostoma*), male preference for conspecific mates appears to arise before female preferences during the early stages of allopatric speciation, and research suggests that male, but not female, preferences become reinforced upon secondary contact. In the current study, we aimed to determine whether the geographically widespread darter species *Etheostoma zonale* exhibits a signature of reinforcement, by comparing the strength of preference for conspecific mates between populations that are sympatric and allopatric with respect to a close congener, *E. barrenense*. We examined the strength of preference for conspecifics for males and females separately to determine whether the preferences of one or both sexes have been strengthened by reinforcement. Our results show that both sexes of *E. zonale* from sympatric populations exhibit stronger conspecific preferences than *E. zonale* from allopatric populations, but that female preferences appear to be more strongly reinforced than male preferences. Results therefore suggest that reinforcement of female preferences may promote behavioral isolation upon secondary contact, even in a genus that is characterized by pervasive male mate choice.

## Graphical abstract

Reinforcement can be an important step in the process of speciation. While many studies of reinforcement focus on female preferences, we found evidence that both female and male preferences show the signature of reinforcement in a sexually dimorphic species of fish. Although both sexes appear to have their preferences shaped by reinforcement, our results suggest that females may face higher costs of maladaptive hybridization than males in this species.

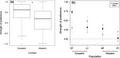

## 1 INTRODUCTION

Behavioral isolation is the reduction in gene flow between species or populations due to divergent mating signals and preferences and is recognized as a powerful reproductive barrier in nature (Coyne & Orr, 2004). While behavioral isolation can arise via both male and female preferences (Lande, 1981; von Schilcher & Dow, 1977), the majority of studies, especially those in sexually dimorphic species, focus on divergence in female mating preferences as the driving force behind isolation (Coyne & Orr, 2004; Panhuis, Butlin, Zuk, & Tregenza, 2001; Ritchie, 2007). This is due, in part, to the general characterization of females as choosy and males as ornamented, competitive, and indiscriminate in their mate choice (Andersson, 1994; Bateman, 1948; Trivers, 1972). However, examples of male mate choice, even in polygynous species, are becoming increasingly common (Bonduriansky, 2001; Edward & Chapman, 2011) with several examples of male mate choice resulting in behavioral isolation between species (Johannesson et al., 2008; Mendelson, Gumm, Martin, & Ciccotto, 2018; Roberts & Mendelson, 2017).

Theoretical models addressing the likelihood of male mate choice suggest that it is evolutionarily constrained relative to female mate choice (Servedio & Lande, 2006). Many evolutionary scenarios model speciation facilitated by the coevolution of female preferences and divergent male traits (e.g., Fisher‐Lande, Lande, 1981; mutation‐order, Mendelson, Martin, & Flaxman, 2014; reinforcement, Servedio, 2007), whereas models of male mate choice show that male preferences are often selected against (Servedio, 2007; Servedio & Dukas, 2013; Servedio & Lande, 2006), especially if male preferences are for “arbitrary” female traits (i.e., traits that do not directly indicate female fecundity; Servedio & Lande, 2006). However, one scenario in which male mate choice may be maintained is in the case of reinforcement (Servedio, 2007).

Reinforcement is the process whereby natural selection against hybrids leads to strengthened behavioral isolation between species (Dobzhansky, 1937; Noor, 1995; Shaw & Mendelson, 2013), with both theoretical and empirical studies showing that reinforcement can play an important role in the process of speciation (Kirkpatrick, 2001; Matute, 2010; Ortiz‐Barrientos, Grealy, & Nosil, 2009; Servedio, 2007; Servedio & Noor, 2003). The classic signature of reinforcement is stronger preferences for conspecifics in populations that are sympatric versus allopatric with respect to a close congener, and many studies investigating reinforcement test for its signature in females (Kelly & Noor, 1996; Kirkpatrick & Servedio, 1999; Lemmon & Lemmon, 2010; Liou & Price, 1994; Servedio & Sætre, 2003). However, empirical support for reinforcement has been found in males for a number of species as well (Gregorio, Berdan, Kozak, & Fuller, 2012; Kronforst, Young, & Gilbert, 2007; Moran & Fuller, 2018; Peterson et al., 2005), and Servedio (2007) found that, under some theoretical conditions, selection on male mate choice is more likely to result in reinforcement than selection on female mate choice. The question therefore remains whether reinforcement is more likely to affect preferences in one sex or the other, and if so, under what conditions.

An effective way to address this question empirically is with comparative studies that quantify male and female preferences for conspecifics across multiple species pairs within a lineage, but these studies are rare. A recent study by Yukilevich and Peterson (2019), for example, found that both male preference and female preference for conspecific mates are widespread in *Drosophila*; however, in species that are sympatric with respect to a close congener, female preferences for conspecifics were stronger than male preferences, suggesting that reinforcing selection primarily acts upon female preferences in sympatry. In contrast, in a study of the darter subgenus *Oligocephalus*, male preferences showed a signature of reinforcement, being stronger for conspecifics in sympatric versus allopatric populations, whereas female preferences for conspecific males were not significant in either sympatric or allopatric populations (Moran & Fuller, 2018). Recent findings also suggest that male preferences for conspecific females evolve earlier than female preferences across several allopatric darter species across the subgenera *Nanostoma* and *Ulocentra* (clade *Simoperca* sensu Near et al., 2011) (Mendelson et al., 2018). In the current study, we focused on two sympatric darter species, from a different subgenus, to evaluate whether a signature of reinforcement was present in males and/or females. Given that reinforcement appears to shape male but not female preferences in *Oligocephalus*, comparing preferences in another darter subgroup may reveal whether this pattern is characteristic of the genus. Specifically, we tested whether (a) preferences for conspecifics are stronger in populations that are sympatric with a close congener than in allopatric populations, that is, the classic signature of reinforcement, and (b) whether one sex exhibits a greater strength of preference for conspecific mates*.*

Darters (Percidae: *Etheostoma*) are a species‐rich group of freshwater fishes native to North America with over 200 described species (Page & Burr, 2011). Premating barriers are found to evolve more rapidly than other barriers in darters (Martin & Mendelson, 2016; Mendelson, 2003; Mendelson, Imhoff, & Venditti, 2007; Williams & Mendelson, 2014), and diversification in darters is thought to occur primarily in allopatry, with sister species pairs all having allopatric ranges (Near et al., 2011). However, natural hybrids have been reported involving over 25% of darter species (Keck & Near, 2009), and recent phylogenetic analyses find evidence of ancient hybridization and genome‐wide introgression between major darter lineages (MacGuigan & Near, 2018). These data suggest that hybridization between darters, both current and historically, is common enough to support a potential role for reinforcement in this system.

**Figure 1:**
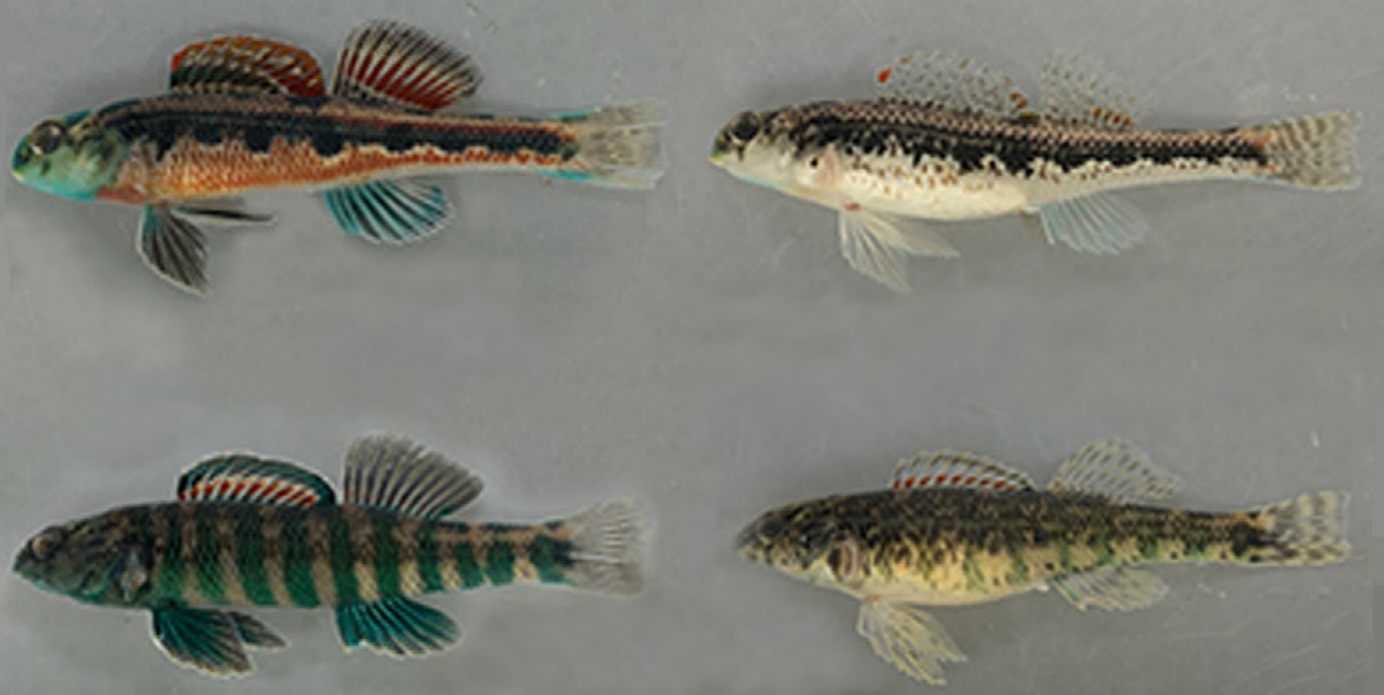
Male and female *E. barrenense* (top row, left and right) and male and female *E. zonale* (bottom row, left and right) with males in breeding coloration

Darter species *Etheostoma zonale* and *E. barrenense* represent some of the most closely related darter species to co‐occur without hybridizing in nature (Hubbs, 1955, 1967; Keck & Near, 2009). Previous work on this species pair shows that crosses between conspecific and heterospecific individuals have similar fertilization and hatching success in the laboratory (Williams & Mendelson, 2014); however, hybrid survivability appears to be reduced relative to that of intraspecific offspring (Williams & Mendelson, 2014), suggesting a degree of postzygotic incompatibility that could promote reinforcement. Male *E. zonale* and *E. barrenense* exhibit elaborate and divergent nuptial coloration and pattern, with male *E. barrenense* displaying primarily red‐orange coloration with black blotches fused along the lateral line, and male *E. zonale* having alternating green and yellow bars along the body (Figure 1). Females of both species display some muted coloration and the patterning that characterizes conspecific males. Previous studies have found that these visual signals are important for mate choice in both females (Williams & Mendelson, 2010) and males (Roberts & Mendelson, 2017).

**Figure 2:**
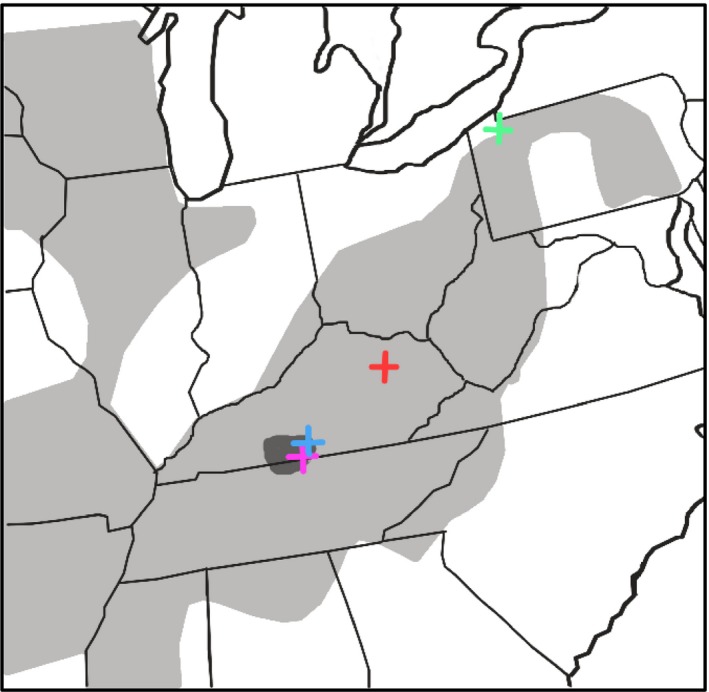
Geographic range of *E. barrenense* (dark gray) and *E. zonale* (light gray) indicating collection sites for the current study. Collection sites are EF = East Fork Barren River (blue), FC = French Creek tributaries (green), MF = Middle Fork Red River (red), LC = Line Creek (purple). Figure modified from Kuehne and Barbour (1983)

For this study, we used *E. zonale* as a focal species since most of its range is allopatric with respect to *E. barrenense* (Figure 2). The geographic range of *E. barrenense* is small and entirely sympatric with *E. zonale,* whereas *E. zonale* occurs over a wide range throughout the Mississippi River basin, Ohio River basin, Ozark‐Ouachita drainage, and was recently (1950s–1960s) introduced into the Susquehanna River (Kneib, 1972). If reinforcement plays a role in the evolution of mate preferences in *E. zonale*, we predicted that populations in areas of sympatry with *E. barrenense* would have stronger preference for conspecific mates than populations allopatric with *E. barrenense*. Additionally, we measured the strength of preference for conspecifics in both males and females to determine whether reinforcement has sex‐specific effects in the focal species.

## 2 METHODS

### 2.1 Overview

To estimate the strength of preference for conspecific mates, we conducted two types of behavioral experiments. Dichotomous mate choice trials gave individual fish a choice between a conspecific or heterospecific of the opposite sex and did not allow fish to physically interact. Artificial stream assays simulated natural conditions and allowed multiple fish of both sexes and species to freely interact. Details of the experimental designs are provided below. Both trial types were conducted previously for a single sympatric population (Roberts & Mendelson, 2017; Williams & Mendelson, 2010). For the current study, we conducted trials with two allopatric populations and an additional sympatric population of *E. zonale* with respect to *E. barrenense*. We combined data from these populations with those from the previously studied sympatric population to test for a signature of reinforcement.

### 2.2 Fish collection and maintenance

We collected *E. zonale* by kick seine from two allopatric populations and one sympatric population for the current study, between 22 March and 26 April 2017. The two allopatric populations were the Middle Fork Red River in Powell Co. Kentucky (hereafter, MF) and tributaries of French Creek in Venango, Crawford, and Erie Co., Pennsylvania (FC). The sympatric population was collected from Line Creek in Clay Co., Tennessee and Monroe Co., Kentucky, on 22 March 2018 (LC). The sympatric population examined in previous studies was from the East Fork Barren River, Monroe Co., Kentucky (EF) (Roberts & Mendelson, 2017; Williams & Mendelson, 2010; Table 1; Figure 2). We also collected *E. barrenense* from this latter site on 23 March 2017 to use as heterospecific stimuli for the allopatric *E. zonale* trials. All sites are part of the Ohio River Basin and contained similar assemblages of darter species (Table S1). Permission to collect fish was granted by the Kentucky Department of Fish and Wildlife Resources (#SC1711121) and the Pennsylvania Fish and Boat Commission (#2017‐01‐0049).

**Table 1:** Collection sites and number of *E. zonale* of each sex collected at each site for dichotomous choice and artificial stream trials. Context (sympatric or allopatric) refers to *E. zonale* with respect to *E. barrenense*

Site	Coordinates	*N* female	*N* male	Context
East Fork Barren River^a^				Sympatric
Monroe Co., KY	36.745964, −85.696728	18	16	
Line Creek				Sympatric
Monroe Co., KY	36.651835, −85.820182	10	22	
Clay Co., TN	36.606639, −85.745970	9	3	
Middle Fork Red River				Allopatric
Powell Co., KY	37.815002, −83.71871	30	19	
French Creek tributaries				Allopatric
Venango Co., PA	41.4200010, −79.8753567	0	1	
Crawford Co., PA	41.522391, −80.049847	18	15	
Crawford Co., PA	41.6951446, −80.1138617	3	2	
Erie Co., PA	41.9613063, −79.9722471	7	1	

a Data for females from Williams and Mendelson (2010) and for males from Roberts and Mendelson (2017).

We transported fish to the University of Maryland Baltimore County and housed them in a recirculating aquarium system (Aquatic Habitats, Inc.). Water temperature, conductivity, and pH for fish housing replicated the natural habitat (temperature = 12°C; conductivity = 550–650 μs; pH = 8.3). We separated individual fish into gravel‐lined aquaria that were visually isolated from individuals of the opposite sex, species, and population and maintained a 12:12‐hr light/dark cycle. Fish were fed a diet of live black worms provided once daily.

### 2.3 Dichotomous choice assays

Dichotomous choice assays followed previously published methods from Roberts and Mendelson (2017) and Williams and Mendelson (2010). Briefly, a 37.9‐liter glass “focal” tank (50*L* × 25*W *× 30*H *cm) was positioned between two 9.6‐L glass “stimulus” tanks (30*L *× 15*W *× 20*H *cm) so that the long sides of the stimulus tanks were flush against the short side of the focal tank. The focal tank was marked with two 5‐cm “association zones” at either end closest to the stimulus tanks. Trials took place between 27 March–2 May 2017 and 27 March–4 April 2018, coinciding with the natural breeding season (Etnier & Starnes, 1993).

The focal and stimulus tanks were lined with gravel of equal heights and filled with equivalent depths of water from the aquarium housing and individually aerated. Each stimulus tank was illuminated with an incandescent light source (GE Crystal Clear, A19, 100W), and a 91 cm full‐spectrum light source (Coralife^®^ F/W/T5 Aqualight, 21 W Colormax™ bulb, 21°W 6700 K bulb) spanned all three tanks. Prior to a trial, an opaque partition was placed between each stimulus tank and the focal tanks, then one *E. barrenense* was placed into a stimulus tank, one *E. zonale* of the same sex was placed in the other, and a focal *E. zonale* (opposite sex of stimulus fish) was introduced into the test tank. Once the focal fish began free‐swimming activity, the opaque partitions were removed and acclimation began. Acclimation was complete after the focal fish entered both association zones and subsequently entered the “neutral zone” (i.e., was not in either association zone). Following acclimation, a 15‐min observation period began, and time spent in each association zone was recorded using JWatcher™ V1.0 (Blumstein, Evans, & Daniel, 2000). Association time appears to be a reliable indicator of mating preference in several species of fishes (Aspbury & Basolo, 2002; Gonçalves & Oliveira, 2003; Jeswiet & Godin, 2011; Lehtonen & Lindström, 2008), including darters (Martin & Mendelson, 2013; Williams & Mendelson, 2010), meaning that association time in the current study is likely a reliable proxy for mate preference. Between trials, gravel was rinsed and mixed, and water was replaced in the test and stimulus tanks. We alternated the side of the test tank in which the conspecific individual was placed to help control for experimental side bias.

We tested the preferences of male and female *E. zonale* from the Middle Fork Red River (MF, allopatric), French Creek (FC, allopatric), and Line Creek (LC, sympatric) populations (*N* = 18 for each sex and population). Stimulus fish for the allopatric MF and FC populations were a conspecific individual from the same site as the focal fish and a heterospecific individual (*E. barrenense*) from the EF population. Stimulus fish for the sympatric LC population were from the same site: a conspecific individual from LC and a heterospecific individual (*E. barrenense*) from LC. Stimulus individuals were size matched within 15% of their standard length (snout to caudal peduncle). A 15% size difference has been shown to be nonsignificant for paired stimuli in females from the EF population (Roberts & Mendelson, 2017), and post hoc analyses for the LC, MF, and FC populations show no significant difference between paired stimuli (paired Wilcoxon signed rank test: *Z* = −0.086, *p* = .93).

### 2.4 Artificial stream assays

Preferences expressed as association time in dichotomous trials may not result in mate choice under more natural, unrestricted conditions (Dougherty & Shuker, 2014), so we also analyzed mate choice of males and females in artificial stream assays, in which fish in breeding condition had full access to one another. Stream assays took place in a Living Stream artificial stream system; model LSW‐700 (Frigid Units Inc.). The stream unit (213*L* × 61*W* × 56*H *cm) contained approximately 530 L of flowing water maintained at a constant temperature of 12°C and matched to the water quality of the aquarium housing. We lined the bottom of the stream with natural rocks and added synthetic structures (foam aquarium filters) known to be attractive spawning substrate for the focal species (TC Mendelson, pers. obs.).

Stream trials for the allopatric MF and FC populations took place between 6 and 16 May 2017; each replicate trial consisted of three *E. zonale* of each sex from one of the allopatric populations and three *E. barrenense* of each sex from the EF population. Three replicate trials consisting of a unique suite of individuals were conducted per allopatric site. One replicate trial for the MF population only consisted of two female *E. zonale*, due to species misidentification (*E. caeruleum*) of the third female. Stream trials for the sympatric LC population took place between 24 and 29 March 2018 and consisted of three individuals of each sex and species per replicate from the LC site. Three replicate trials with unique individuals were conducted for LC. For all artificial stream trials, fish were size matched within 15% of their standard length to at least one heterospecific individual of the same sex. Fishes were used between 1 and 2 times within population replicates due to limitations in individuals of an appropriate size to meet size‐matching criteria, but every replicate consisted of a unique combination of individuals.

The artificial stream was lit with a full spectrum (Coralife^®^ F/W/T5 Aqualight, 21 W Colormax™ bulb, 21°W 6700 K bulb, Energy Savers Unlimited, Inc.) and two incandescent (GE Crystal Clear, A19, 100 W) lights. Test groups acclimated to the artificial stream for 35 min, after which time most individuals exhibited typical swimming behavior (pers. obs.). Following acclimation, we began a six‐hour observation period during which we tallied five types of behavior for both *E. zonale* and *E. barrenense*: (a) *spawning events*, defined by the rapid and synchronized quivering of a male–female pair, followed by a brief contact of the genital regions to the substrate (indicating the release of gametes); (b) *successful male solicitation*, defined by a male's approach toward a stationary female followed by his periodic body twitching alongside the female, but where the release of gametes was not observed by either sex; (c) *unsuccessful male solicitation*, defined as a male's approach toward a stationary female and the female's subsequent fleeing; (d) *chases between males*, defined as a male's approach toward another male resulting in accelerated pursuit and fleeing by the respective males; and (e) *male chases of females*, defined as a male changing his direction or speed in pursuit of a swimming female. Trials began between 900 and 1,100 hr. Both the acclimation time and entire trial time were recorded by two Panasonic HX‐A1 Action Cam's (Panasonic Corp.), each situated to record one half of the artificial stream unit. Video of each trial was used to score behaviors by one researcher (NSR).

### 2.5 Statistical analysis

Strength of preference (SOP) was calculated for each individual using the equation:

(1) $$\text{SOP}=\left(T_{C}-T_{H}\right)/\left(T_{C}+T_{H}\right)$$

where *T*_C_ is the total time spent in the conspecific association zone and *T*_H_ is the total time spent in the heterospecific association zone over the entire 15‐min trial. SOP can range from +1 to −1 indicating a complete preference for conspecific or heterospecific individuals, respectively, whereas a score of 0 indicates no preference. This measure of preference represents the proportional reduction in gene flow relative to expectations under random mating (e.g., a value of 0.3 is interpreted as a 30% reduction in gene flow due to behavioral isolation than expected under random mating; Sobel & Chen, 2014).

We used a general linear model to determine which factors, or interactions of factors, explain variation in SOP in dichotomous trials. The main effects we examined were context (i.e., sympatry or allopatry), population (i.e., EF, LC, MF, or FC), sex, and interactions of these terms. Population was nested within context in all models since these two factors exhibit hierarchical data structure (i.e., population is a smaller spatial scale encompassed by context). Model selection started with a full model, including all possible terms and interactions. We then sequentially removed nonsignificant variables from the full model to reduce model parameters. Our final model included all single variables (i.e., context, sex, and population) and the interaction of sex and population as explanatory variables for SOP. Post hoc analyses compared strength of preference between context and populations using least squared means with a Bonferroni adjustment for multiple comparisons and Mann–Whitney *U* tests to analyze data from male and female *E. zonale* independently.

For the artificial stream trials, isolation indices (*I*) were calculated separately for both *E. zonale* and *E. barrenense* after Stalker (1942) for each of the five behaviors recorded (spawning, successful solicitation, unsuccessful solicitation, male–male chase, male–female chase) as

(2) $$I=\frac{\left(\begin{array}{c} \text{mean no}\text{. conspecific interactions across replicates}\\ \;-\;\text{mean no}\text{. heterospecific interactions across replicates} \end{array}\right)}{\left(\begin{array}{c} \text{mean no}\text{. conspecific interactions across replicates}\\ +\text{mean no}\text{. heterospecific interactions across replicates} \end{array}\right)}$$

Isolation indices for the first five behaviors are available for the previously reported EF population, based on the same equation. However, artificial stream trials in that study (Williams & Mendelson, 2010) used slightly different methods than the present study, with only one species of female in each trial. Isolation indices from the EF population therefore are reported separately, for comparison.

Additionally, a total isolation index, taking into account all five behaviors together, was calculated for the artificial stream assays in the current study. For each replicate, count data for the five behaviors were summed, for each species separately, to yield the total number of conspecific‐directed behaviors and the total number of heterospecific‐directed behaviors in each replicate. We took the average of these summed totals across the three replicates per population and applied these values to Equation 2 to calculate a total isolation index for each species and population.

We used a generalized least squared (GLS) model to determine the effect of context (sympatry or allopatry), species, and population on the total isolation index calculated for the artificial stream assays for the LC, MF, and FC populations. Using a GLS allowed us to account for heteroskedastic data (Zuur, Ieno, Walker, Saveliev, & Smith, 2009). All *E. barrenense* were coded as sympatric with respect to *E. zonale*, while *E. zonale* were coded as sympatric or allopatric accordingly. Model selection started with a full model, including all possible terms and interactions, and included trial replicate as a random effect. We then sequentially removed nonsignificant terms to select the fixed effect structure of the final model. The final model included only context (sympatry or allopatry) as explanatory variables for total isolation. All analyses were conducted in R (ver. 3.5.0; R Core Development Team, 2015). Post hoc comparisons were calculated using the package “lsmeans.”

## 3 RESULTS

### 3.1 Dichotomous choice trials

Male and female *E. zonale* from both sympatric populations had mean ± *SE* SOP significantly greater than zero, indicating a significant preference for conspecifics. SOP for females from the EF population (data from Williams & Mendelson, 2010) was 0.73 ± 0.03 (two‐tailed *t* test: *t*_17_ = 18.79,* p* = 8.30 × 10^−13^), and SOP for males from the EF population (data from Roberts & Mendelson, 2017) was 0.30 ± 0.14 (*t*_15_ = 2.16,* p* = .048). SOP for females from the LC population was 0.31 ± 0.15 (*t*_17_ = 2.11, *p* = .049), and SOP for males from the LC population was 0.48 ± 0.12 (two‐tailed Wilcoxon signed rank test: *Z* = 2.92, *p* = .003). From the allopatric populations, SOP was lower, and it was significantly different from zero only for female *E. zonale* from the MF population; SOP = 0.27 ± 0.12 (*t*_17_ = 2.19, *p* = .043). SOP for male *E. zonale* from MF was 0.11 ± 0.15 (*t*_17_ = 0.75, *p* = .46). SOP for females from FC was 0.02 ± 0.10 (*t*_17_ = 0.23, *p* = .82), and SOP for males from FC was 0.20 ± 0.13 (*t*_17_ = 1.52, *p* = .15).

**Figure 3:**
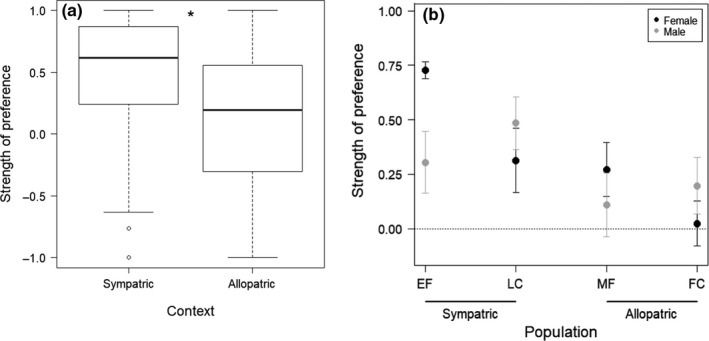
Strength of preference for conspecific mates for *E. zonale* in dichotomous mate choice trials, from populations sympatric and allopatric with respect to *E. barrenense* for (a) combined sexes and populations within context and (b) separated by sex and population. Positive strength of preference indicates a preference for conspecifics of the opposite sex, while negative values indicate a preference for heterospecifics of the opposite sex. (a) Bars represent medians, boxes indicate upper and lower quartiles, whiskers show sample minima and maxima, and open circles show outliers. * Indicates a significant difference between groups (least square means: *p* = .006). (b) Mean ± *SE* for East Fork Barren River = EF, Line Creek = LC, Middle Fork Red River = MF, and French Creek tributaries = FC. Data for females from the EF population are from Williams and Mendelson (2010) and for males from the EF population from Roberts and Mendelson (2017)

The results of our final model for the dichotomous choice trials showed that context and the interaction of sex and population were significant factors in predicting strength of preference (Table 2). Model results indicate that *E. zonale* from populations sympatric with *E. barrenense* had significantly greater preferences for conspecific mates than *E. zonale* from allopatric populations (Table 2; Figure 3a). A post hoc *t* test comparing SOP between sympatric and allopatric populations also revealed that SOP was significantly greater for sympatric than for allopatric populations (*t* = −3.53, *p* = .0006; Figure 3a). The final model also showed a small but significant effect of the interaction of population and sex (Table 2; Figure 3b). This appears to arise from a significant difference in SOP between the sympatric populations for female *E. zonale* (EF–LN, *t*_134_ = 2.40, *p* = .02). There was no significant difference in SOP between the allopatric populations for females (MF–FC, *t*_134_ = –1.43, *p* = .15), and no significant difference in SOP for males between the two allopatric populations or the two sympatric populations (EF–LC, *t*_134_ = −1.01, *p* = .31; MF–FC, *t*_134_ = 0.51, *p* = .61).

**Table 2:** Results of the linear model for SOP of *E. zonale* from dichotomous choice assay

Source	*df*	*F* value	*p* value
**Context**	1	12.835	**.0005**
Sex	1	0.424	.516
Population (nested within context)	2	0.749	.475
**Population x Sex interaction**	3	2.709	**.048**
Error	134		

Note

Significant factors are shown in bold.

**Figure 4:**
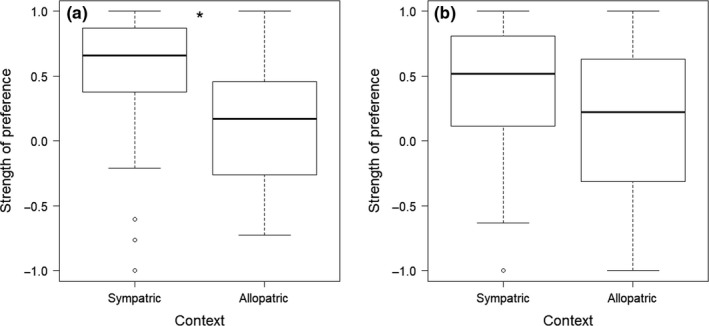
Strength of preference for conspecific mates in populations of *E. zonale* that are sympatric and allopatric with respect to *E. barrenense,* for (a) females and (b) males in dichotomous mate choice trials. Strength of preference is significantly greater in sympatric populations than allopatric populations for females (*p* = .0004), but not males (*p* = .06), although the data trend in the expected direction given a hypothesis of reinforcement. Bars represent medians, boxes indicate upper and lower quartiles, whiskers show sample minima and maxima, and open circles show outliers. *Indicates a significant difference between groups (Mann–Whitney *U* test)

Notably, there was no significant effect of sex or population on strength of preference (Table 2), suggesting that the signature of reinforcement is not more evident in one sex or the other. A post hoc analysis of each sex separately, however, combining populations within geographic context, showed that sympatric females had significantly greater SOP than allopatric females (sympatry: 0.52 ± 0.08; allopatry: 0.15 ± 0.08; Mann–Whitney *U* test: *Z* = −3.49,* p* = .0004; Figure 4a), whereas this comparison was not significant for males (sympatry: 0.40 ± 0.09; allopatry: 0.15 ± 0.10; *Z* = −1.88, *p* = .06; Figure 4b). Further, using Cohen's *d* (Cohen, 2013) to determine the effect size of context on SOP showed a medium effect of context for females (*d* = 0.75) and a small effect for males (*d* = 0.44).

### 3.2 Artificial stream trials

**Figure 5:**
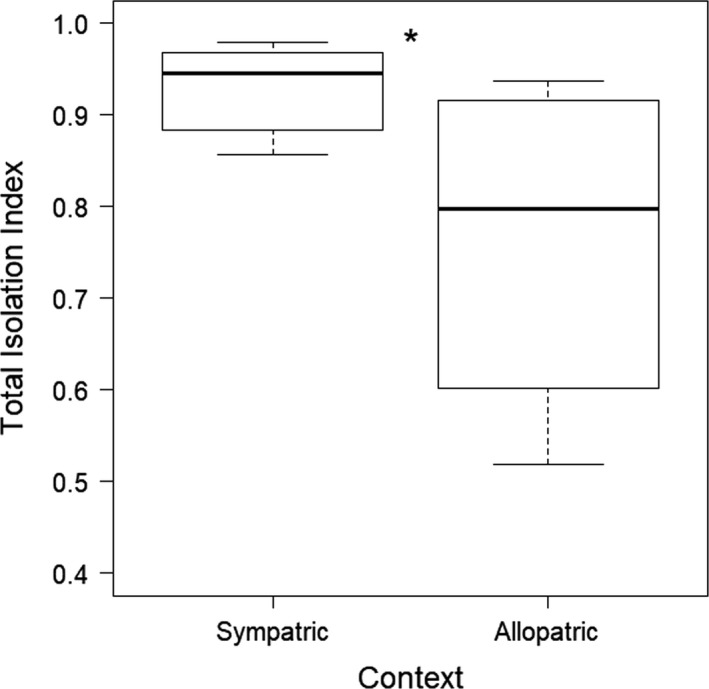
Total isolation as measured in artificial stream trials. The total isolation index ranges from +1 to −1, with positive values indicating a conspecific bias and negative values indicating a heterospecific bias. Bars represent medians, boxes indicate upper and lower quartiles, whiskers show sample minima and maxima, and open circles show outliers. All *E. barrenense* were coded as sympatric, and *E. zonale* was coded as sympatric or allopatric accordingly (sympatric *N* = 12; allopatric *N* = 6). *Indicates a significant difference between groups (GLS: *p* = .04)

The final model found a significant effect of context (i.e., sympatry/allopatry) on total isolation in the artificial stream trials (GLS: *t* = 2.25, *p* = .04; Figure 5). Results of model validation found no significant effect of species or interactions between context and species, meaning that total isolation was not explained by one species or the other.

**Figure 6:**
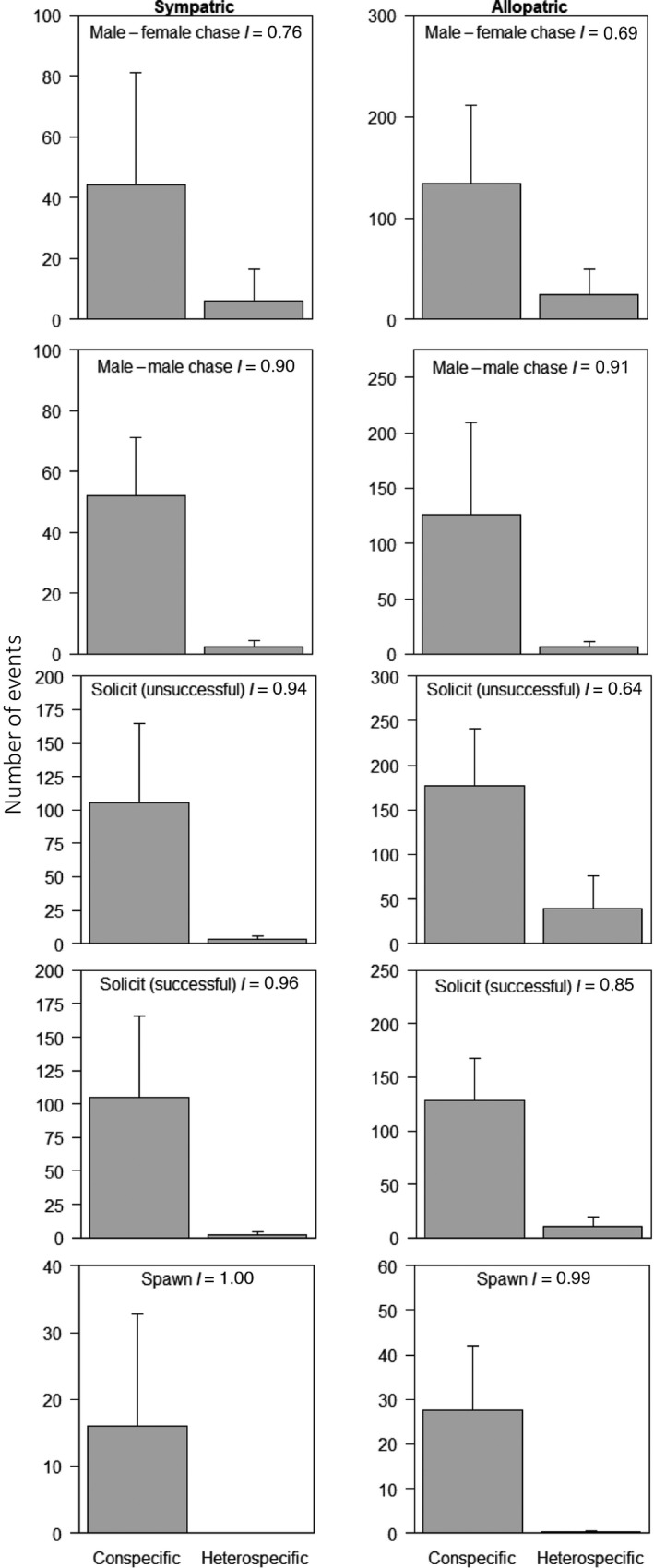
Behavioral interactions between conspecific and heterospecific individuals in artificial stream trials for *E. zonale*. Mean and standard deviation for populations of *E. zonale* sympatric (left‐hand column) and allopatric (right‐hand column) with respect to *E. barrenense*, for male–female chases, male–male chases, unsuccessful and successful solicits, and spawning events. Isolation indices (*I*) for each behavior are shown, with possible values ranging from + 1, indicating a complete conspecific bias, to −1, indicating a complete heterospecific bias

Isolation indices for both *E. zonale* and *E. barrenense* were all positive, indicating conspecific bias, and ranged from 0.43 to 1.00 (Table 3). Isolation indices were lower for populations of *E. zonale* allopatric with respect to *E. barrenense* than for sympatric *E. zonale* across all recorded behaviors except male–male chases (Figure 6). Interspecific spawning was only observed once throughout all trials and was a result of a group spawn in which a male *E. zonale* from the allopatric MF population spawned alongside a pair of spawning *E. barrenense*. Thus, the difference in isolation indices between sympatric and allopatric populations is largely accounted for by differences in male solicitation behaviors (both successful and unsuccessful), with males from allopatric populations more likely to court heterospecific females. Indeed, removal of male solicitation behaviors from the final model resulted in no significant difference between sympatric and allopatric populations in total isolation index (GLS: *t* = −0.60, *p* = .56).

**Table 3:** Average and standard deviations of conspecific‐ and heterospecific‐directed behaviors for artificial stream assays

Trial	Pop.	Species	Male–female chase			Male–male chase			Solicit (unsuccessful)		
			Conspecific	Heterospecific	*I*	Conspecific	Heterospecific	*I*	Conspecific	Heterospecific	*I*
Sympatric^a^	EF	*E. barrenense*	–	–	–	134.0 ± 79.0	15.3 ± 20.5	0.80	393.0 ± 134.0	0	1.00
	EF	*E. zonale*	–	–	–	58.7 ± 39.4	9.7 ± 17.0	0.72	237.0 ± 27.7	0	1.00
Sympatric	LC	*E. barrenense*	34.0 ± 44.8	7.7 ± 9.9	0.63	114.3 ± 111.8	1.0 ± 1.0	0.98	78.0 ± 37.3	4.0 ± 3.5	0.90
	LC	*E. zonale*	44.3 ± 36.7	6.0 ± 10.4	0.76	52.0 ± 19.1	2.3 ± 2.1	0.91	105 ± 59.6	3.0 ± 2.6	0.94
Allopatric	EF	*E. barrenense*	97.3 ± 56.7	8.0 ± 5.3	0.85	146.0 ± 79.6	0.7 ± 0.6	0.99	84.0 ± 35.5	4.0 ± 3.5	0.91
	FC	*E. zonale*	102.3 ± 28.5	7.3 ± 3.2	0.87	76.0 ± 65.0	9.0 ± 5.6	0.79	193.0 ± 44.7	14.7 ± 9.6	0.86
Allopatric	EF	*E. barrenense*	185.0 ± 187.4	8.0 ± 13.0	0.92	66.0 ± 64.1	2.0 ± 1.0	0.94	176.3 ± 195.1	1.0 ± 1.0	0.99
	MF	*E. zonale*	165.0 ± 105.1	41.7 ± 25.8	0.60	176.3 ± 73.2	3.7 ± 0.6	0.96	162.0 ± 85.0	63.0 ± 40.9	0.44

Trial	Population	Species	Solicit (successful)			Spawn			Total Isolation		
			Conspecific	Heterospecific	*I*	Conspecific	Heterospecific	*I*	Conspecific	Heterospecific	*I*_Total_
Sympatric	EF	*E. barrenense*	72.0 ± 24.0	0	1.00	6.0 ± 10.0	0	1.00	605 ± 157.7	15.3 ± 20.5	0.95
	EF	*E. zonale*	107.0 ± 36.0	2.0 ± 3.5	0.95	17.7 ± 20.4	0	1.00	420.4 ± 63.5	11.7 ± 17.4	0.95
Sympatric	LC	*E. barrenense*	108.7 ± 113.1	2.3 ± 3.2	0.96	26.0 ± 22.6	0	1.00	361 ± 207.0	15.0 ± 12.3	0.92
	LC	*E. zonale*	105 ± 60.6	2.0 ± 2.6	0.96	16.0 ± 16.8	0	1.00	322.3 ± 184/0	13.3 ± 16.3	0.92
Allopatric	EF	*E. barrenense*	126.3 ± 23.3	1.7 ± 1.2	0.97	61.0 ± 40.0	0	1.00	514.7 ± 134.8	14.3 ± 8.0	0.95
	FC	*E. zonale*	119.7 ± 58.1	7.0 ± 4.6	0.89	22.3 ± 20.5	0	1.00	513.3 ± 191.3	38.0 ± 22.5	0.86
Allopatric	EF	*E. barrenense*	40.7 ± 24.1	4.7 ± 2.1	0.79	31.7 ± 26.7	0	1.00	499.7 ± 394.3	15.7 ± 13.3	0.94
	MF	*E. zonale*	136.7 ± 18.4	13.7 ± 13.0	0.82	33.0 ± 1.7	0.3 ± 0.6	0.98	673.0 ± 166.3	122.3 ± 75.4	0.69

Note

Total isolation represents the sum of all conspecific‐ and heterospecific‐directed behaviors across all measured behaviors.

Abbreviations: EF, East Fork Barren River; FC, French Creek tributaries; LC, Line Creek; MF, Middle Fork Red River.

a Data for EF sympatric population from Williams and Mendelson (2010).

## 4 DISCUSSION

Our aim was to evaluate geographic variation in mate choice in *Etheostoma zonale* and determine whether it is consistent with a pattern of reinforcement. We also aimed to determine whether a pattern of reinforcement is more evident in males than females, as was shown in a previous study in the *Oligocephalus* subgenus of *Etheostoma* (Moran & Fuller, 2018). Our results were consistent with a pattern of reinforcement. We found that *E. zonale* from populations sympatric with the close congener *E. barrenense* had a greater preference for conspecific mates than *E. zonale* from allopatric populations when tested in dichotomous choice trials (Figure 3a). Results of our artificial stream assays further support this finding, with geographic context being the only significant predictor of total isolation and overall stronger isolation indices in sympatric versus allopatric populations (Figure 5). This signature of reinforcement—greater preference for conspecific mates in populations that are sympatric with a close congener compared to allopatric—was evident for both males and females in the dichotomous mate choice trials in our linear model, but was statistically significant only for females when evaluating each sex independently (Figure 4). Our results therefore suggest that reinforcement acts on both male and female preferences in *E. zonale*, but they do not support a universally stronger effect of reinforcement on males in the genus *Etheostoma.*

Previous studies that address reinforcement in both sexes provide conflicting support for whether male or female preferences are more strongly affected. One study in *Drosophila* found that while male and female preferences were widespread, female preferences were primarily responsible for isolation between species in sympatry (Yukilevich & Peterson, 2019). That result is consistent with basic sexual selection theory, which assumes that females invest more heavily in reproduction than males and would pay a higher cost for maladaptive mate choice (Andersson, 1994). However, in *Etheostoma caeruleum* and the *E. spectabile* species complex (all in the darter subgenus *Oligocephalus*)*,* males have both heightened conspecific mate preference and aggression bias in sympatric as compared to allopatric populations (Moran & Fuller, 2018). In this same group, female preference for conspecific males was determined to be insignificant in both sympatric and allopatric populations (Moran & Fuller, 2018; Moran, Zhou, Catchen, & Fuller, 2017). Combined with an additional study showing maladaptive effects of hybridization in sympatric species pairs in this subgenus (Moran, Zhou, Catchen, & Fuller, 2018), their results suggest that selection is acting on male, but not female, mate choice in sympatric populations. Our results, which taken together suggest that female preferences are more strongly reinforced in sympatric populations, are therefore more consistent with results from *Drosophila* than with those of a congeneric species group. 

Several notable differences between our focal species pair and species tested by Moran and Fuller (2018) could account for the disparity between the studies. One difference is methodological, in that the dichotomous trials of Moran and Fuller (2018) allowed unrestricted access among two males and one female. An unrestricted design allows mate choice to be quantified in a more naturalistic setting, but it can mask female preference if male behavior has a greater effect on mating outcomes (Dougherty & Shuker, 2014). Estimating female preference in unrestricted choice trials therefore must control for male behavior, essentially quantifying the degree to which females mate with males more than expected given male courtship intensity (see also Martin & Mendelson, 2016). In the current study, female preference in the dichotomous choice trials, where males are restricted from interacting with each other and with the focal female, may be less likely confounded by male behavior. Thus, one explanation for the difference between the two studies in the effect of sympatry on female preference is that female preference was measured in different ways.

Another difference between the studies is the biology of the focal species. Whereas males of *E. zonale* and *E. barrenense* differ markedly in nuptial coloration—green vertical bars compared to a black horizontal stripe on a vivid red background (Figure 1)—species used in Moran and Fuller (2018) are characterized by similar male coloration, that is, alternating red and blue vertical bars (e.g., Page & Burr, 2011). Ichthyologists can identify distinguishing species‐specific color features, but the differences are subtle compared to those of the focal species in our study (e.g., Page & Burr, 2011). Interestingly, previous studies of species in the subgenus *Oligocephalus* have consistently failed to find female preferences for male color patterns either within or between species (Fuller, 2003; Moran & Fuller, 2018; Moran et al., 2017; Pyron, 1995; Zhou, Loew, & Fuller, 2015). Rather, isolation is thought to be maintained by male behaviors in *Oligocephalus,* with males preferring conspecific over heterospecific females (Moran & Fuller, 2018; Moran et al., 2017; Zhou et al., 2015). There is also evidence that male coloration functions as an aggressive male–male signal in competition over access to females as opposed to being a target of female selection in that group (Moran et al., 2017; Zhou & Fuller, 2016). In contrast, females in the focal species pair have been shown to prefer conspecific over heterospecific nuptial hue (red vs. green), nuptial pattern (bars vs. stripe), and hue and pattern combined (Williams & Mendelson, 2011). Female *E. barrenense* also prefer one hue of conspecific red over another, providing evidence of female preference for color variation within species as well (Williams, Gumm, & Mendelson, 2013). Thus, female preference for male nuptial color may be present in some, but not all, species of darters. In these species, female preferences may be shaped by reinforcement. Further, the focal species in our study diverged approximately 6.5 mya (Williams & Mendelson, 2010), whereas *E. spectabile* and *E. caeruleum* diverged approximately 22 mya (Near et al., 2011), suggesting that the two studies may represent different time scales of evolutionary change.

One important result of our study was that the strength of preference for conspecifics differed between the two sympatric populations, such that the signature of reinforcement we observed for females in the dichotomous mate choice trials was only evident in one of the sympatric populations. Population level effects have been demonstrated in other studies of reinforcement; however, these differences are usually related to distance from sympatry, with conspecific preference decreasing as distance from sympatry increases (Gabor, Ryan, & Morizot, 2005; Gregorio et al., 2012; Kronforst et al., 2007). There was no significant variation in SOP between allopatric populations of *E. zonale* despite large geographical distances between the two allopatric sites. This suggests that there is no clinal effect of reinforcement in *E. zonale,* at least over the geographical scale that we tested. Nonetheless, our results highlight the importance of testing multiple populations in studies of mate choice and reinforcement.

Although the effect of sex on SOP was not significant in our linear model, the post hoc analysis of male and female data independently found that male preference for conspecific females was not significantly stronger in sympatric versus allopatric populations, although the trend (*p* = .06) was in the expected direction given a hypothesis of reinforcement; thus, male preferences likely contribute to behavioral isolation in sympatry, but may not be reinforced to the same extent as female preferences. Recently, Mendelson et al. (2018) simulated secondary contact among closely related, allopatric populations (species) of *Etheostoma,* and found that male preferences for conspecifics were consistently stronger than female preferences. That result, and those of others examining the *Oligocephalus* subgenus (Moran & Fuller, 2018; Moran et al., 2017; Zhou et al., 2015), suggests that the coevolution of female preferences and male ornaments may not best explain the earliest stages of behavioral isolation in *Etheostoma* and reinforcement may play an important role. There is no evidence of hybridization between the focal species pair in nature (Hubbs, 1955, 1967), and a previous study indicates a potential reduction in F1 hybrid survival (Williams & Mendelson, 2014). Our results therefore warrant further investigation into sex‐specific fitness costs to hybridization for this species pair.

In addition to dichotomous choice assays, which allowed us to separately test the strength of male and female preferences, artificial stream assays allowed us to observe courtship and spawning behaviors in freely interacting individuals. We found that trials of sympatric populations yielded greater total isolation indices than trials with *E. zonale* from allopatric populations (Figure 5). The difference in total isolation between sympatric and allopatric trials appears to be accounted for by differences in male solicitation behaviors. Indeed, interspecific spawning events were rare even in allopatric trials, with only one instance observed across all trials (Table 3; Figure 6). Because all *E. barrenense* are sympatric with respect to *E. zonale*, it is possible that spawning did not occur in either sympatric or allopatric trials due to female *E. barrenense* rejecting a heterospecific (*E. zonale*) male and male *E. barrenense* failing to court heterospecific females. A reduction in solicitation of heterospecifics by male *E. zonale* in sympatric stream trials would seem inconsistent with post hoc analysis of the dichotomous mate choice trials, in which male preference for conspecifics did not vary significantly with geographic context. However, the near significant trend (*p* = .06) for stronger male preference in sympatry in dichotomous trials is consistent with the lack of significant effect of sex in the model results and suggests that male preference for conspecifics may have manifest in the more naturalistic stream trials.

Isolation indices from the artificial stream assays for the sympatric population in the current study (LC) are comparable to those for the sympatric population reported in the previous study (EF). For comparison, isolation indices for the EF population (Williams & Mendelson, 2010) are presented in Table 3. The largest differences between the previously studied and the current sympatric populations are between male–male chase behaviors and unsuccessful male unsuccessful courtship attempts. In the current study, there was stronger conspecific bias (greater *I*) in male–male aggression and more unsuccessful heterospecific solicitations (lower *I*) than in the previous study. This difference may be due to differences in experimental design. Most notably, only one species of female was present with males of both species in each of the artificial stream assays in the previous study (Williams & Mendelson, 2010), while the current study had both species of males and females included within each replicate for artificial stream assays. Authors noted that the heterospecific males (relative to the female group) exhibited minimal social interactions compared to the conspecific males, often attempting to escape the stream arena. In the current study, males and females of both species were included in artificial stream assays, and we did not observe either species consistently exhibiting asocial or escape behaviors, which may have increased the likelihood of heterospecific solicitations, albeit unsuccessful, in our experimental design.

In conclusion, we found stronger overall preference for conspecific mates in populations of *Etheostoma zonale* that are sympatric versus allopatric with respect to *E. barrenense.* This classic signature of reinforcement suggests that speciation can be completed in darters by selection for mate choice in sympatry. Reinforcement can shape both male and female preferences (Servedio, 2007), but few studies of reinforcement directly compare both sexes, often focusing exclusively on female preferences, especially in sexually dimorphic species. We found that preferences for conspecifics may be strengthened in sympatry for both males and females in the focal species pair due to reinforcement. Female preferences appear to be more strongly reinforced than male preferences, and previous studies of female choice for male nuptial coloration in these species suggest this could be due to reinforcement of female preference for elaborate male ornaments (Williams et al., 2013; Williams & Mendelson, 2011). In other darter species, however, behavioral isolation is thought to be maintained and reinforced primarily through male choice and male competition (Fuller, 2003; Moran & Fuller, 2018; Moran et al., 2017; Zhou et al., 2015). Given that the prevalence of male mate choice is becoming more broadly studied across animal taxa (Edward & Chapman, 2011) and appears pervasive in the sexually dimorphic genus *Etheostoma* (Ciccotto, Gumm, & Mendelson, 2013; Mendelson et al., 2018; Moran & Fuller, 2018; Moran et al., 2017; Roberts & Mendelson, 2017; Zhou et al., 2015), future work should consider how both male and female preferences contribute to speciation in sympatry and allopatry.

## CONFLICT OF INTEREST

The authors declare no competing interests.

## AUTHOR CONTRIBUTIONS

NSR and TCM conceived the study and conducted field collections. NSR conducted dichotomous choice and artificial stream trials and conducted all statistical analyses. The manuscript was written by NSR with input from TCM.

### Open Research Badge

This article has earned an Open Data Badge for making publicly available the digitally‐shareable data necessary to reproduce the reported results. The data is available at https://doi.org/10.5061/dryad.18931zcsg.

## Data Availability

Data for dichotomous choice assays, artificial stream trials, and all supporting R code are available on Dryad: https://doi.org/10.5061/dryad.18931zcsg.
