# Supplementary material for: Reinforcement in the banded darter Etheostoma zonale: The effect of sex and sympatry on preferences
Source: Ecol Evol. 2020 Feb 12;10(5):2499–512. doi: 10.1002/ece3.6076 (PMC7069321; doi:10.1002/ece3.6076)
Supplement: Supplementary file 1 [file ECE3-10-2499-s001.docx]

Table S1. *Etheostoma* species observed at collection sites.

| Site | Species observed |
| --- | --- |
| East Fork Barren River | *E. barrenense* |
|  | *E. blennioides* |
|  | *E. caeruleum* |
|  | *E. flabellare* |
|  | *E. spectabile* |
|  | *E. stigmaeum* |
|  | *E. zonale* |
| Line Creek | *E. barrenense*  *E. blennioides*  *E. caeruleum*  *E. flabellare*  *E. nigrum*  *E. stigmaeum*  *E. zonale* |
| Middle Fork Red River | *E. baileyi* |
|  | *E. blennioides* |
|  | *E. caeruleum* |
|  | *E. flabellare* |
|  | *E. nigrum* |
|  | *E. variatum* |
|  | *E. zonale* |
| French Creek tributaries | *E. blennioides* |
|  | *E. caeruleum* |
|  | *E. flabellare* |
|  | *E. nigrum* |
|  | *E. variatum* |
|  | *E. zonale* |
